# Beyond the Scale: Changes in Pain, Physical Function (WOMAC), and Low-Grade Inflammation Following Semaglutide Treatment in Patients with Knee Osteoarthritis—Results from a Six-Month Real-World Cohort

**DOI:** 10.3390/jcm15155876

**Published:** 2026-07-27

**Authors:** Alessandro Conforti, Linda Lucchetti, Francesca Ciampa, Marco Bonifacio, Marco Giuseppe Musorrofiti, Valerio Cipolloni, Filippo Messina

**Affiliations:** 1 Rheumatology, ASL Roma 4, 00053 Civitavecchia, Italy; 2 Fisioterapia Medica srl, 00053 Civitavecchia, Italy; 3 Faculty of Health Sciences, Università Telematica eCampus, 22060 Novedrate, Italy; marcomusorrofiti@gmail.com; 4 Department of Orthopaedics, A.O.U. “Vanvitelli” University Hospital, “Luigi Vanvitelli” University, 80100 Naples, Italy

**Keywords:** semaglutide, knee osteoarthritis, obesity, inflammation, synovitis, macrophages, response heterogeneity, clinical phenotypes, cluster analysis, real-world evidence

## Abstract

**Background**: Knee osteoarthritis (KOA) in people with overweight or obesity is clinically heterogeneous, reflecting mechanical loading, synovial and systemic inflammation, metabolic dysfunction, structural severity, and pain-related factors. We evaluated multidomain outcomes after semaglutide initiation and characterized clinically relevant response patterns. **Methods**: This retrospective, self-controlled cohort included 93 adults treated in routine rheumatology care; 88 completed a six-month follow-up. There was no untreated concurrent comparator; within-patient changes and exposure–outcome associations were therefore interpreted as non-causal. Changes in pain, Western Ontario and McMaster Universities Osteoarthritis Index (WOMAC) function, anthropometry, inflammatory markers, glycated hemoglobin (HbA1c), lipids, dose exposure, and safety were evaluated. Joint multivariable models included percentage weight loss and maximum achieved dose. Exploratory K-means phenotyping integrated dose, weight loss, body mass index (BMI) decrease, visual analog scale (VAS) and WOMAC improvement, and C-reactive protein (CRP), erythrocyte sedimentation rate (ESR), and HbA1c reductions, with candidate solutions and resampling stability examined. **Results**: At six months, mean weight decreased by 10.88 kg, VAS pain score by 2.47 points, WOMAC total by 22.50 points, CRP by 2.67 mg/L, ESR by 7.62 mm/h, and HbA1c by 0.51 percentage points (all *p* < 0.001). Weight loss remained independently associated with WOMAC improvement, while achieved dose retained positive adjusted associations with pain, function, inflammatory, and metabolic changes. Exploratory clustering identified five clinically interpretable patterns along a broader response-intensity gradient, including high-burden multidomain response, high-dose concordant response, dose-modified intermediate response, inflammation-preserved response despite moderate weight loss, and dose-limited graded response. These observational coefficients and clusters cannot distinguish treatment-related effects from residual confounding or co-interventions. **Conclusions**: Substantial within-patient improvements were observed, but the uncontrolled design precludes causal attribution. The exploratory patterns may be compatible with contributions from baseline disease burden, dose exposure, mechanical unloading, and residual inflammatory variation; they do not establish weight-independent pharmacologic effects or validated patient subtypes. Prospective controlled, multicenter validation is required.

## 1. Introduction

Knee osteoarthritis (KOA) is a major cause of chronic pain, impaired mobility, and functional limitation. Its clinical expression is not determined by cartilage loss alone: structural damage, synovial inflammation, pain sensitization, muscle weakness, mechanical loading, sleep and mood disturbance, and metabolic comorbidity interact to determine disability. Overweight and obesity are therefore important modifiable contributors, because excess body mass increases compressive forces across the knee while adipose tissue promotes systemic low-grade inflammation. Current international guidance emphasizes education, exercise, weight management, and individualized non-surgical care for people with KOA and excess weight [1,2].

The obesity–KOA relationship is both mechanical and immunometabolic. Adipokines, insulin resistance, dyslipidemia, cytokine signaling, and obesity-associated immune-cell activation can alter the synovial microenvironment, increase nociceptive signaling, and promote cartilage matrix catabolism. Intensive diet and exercise interventions improve pain and function in many patients, but the magnitude of benefit is variable [3]. Real-world analgesic studies by Conforti and colleagues have likewise shown that symptomatic response in KOA is heterogeneous and that routine-care evaluation should include clinically meaningful pain, function, tolerability, and follow-up outcomes rather than a single endpoint [4,5].

Glucagon-like peptide-1 receptor agonists (GLP-1RAs) have transformed the treatment of obesity and type 2 diabetes. Semaglutide at a dose of 2.4 mg once weekly produces substantial weight loss and cardiometabolic improvement [6], and exploratory Semaglutide Treatment Effect in People with obesity (STEP) analyses demonstrated reductions in C-reactive protein (CRP) [7]. STEP 9 subsequently showed greater reductions in body weight and KOA-related pain with semaglutide than with placebo [8]. However, trials and cohort studies with GLP-1RAs have not produced identical pain findings [9,10], suggesting that response may depend on baseline disease, inflammatory burden, exposure, co-interventions, and patient phenotype.

Preclinical evidence provides biological plausibility for effects beyond mechanical unloading. GLP-1 receptor activation has been associated with reduced inflammatory mediator release, macrophage repolarization, inhibition of nuclear factor-kappa B signaling, protection from endoplasmic reticulum stress, and reduced cartilage matrix catabolism in osteoarthritis models [11,12,13]. Obesity itself can induce pro-inflammatory synovial macrophage infiltration before overt cartilage degeneration, while impaired efferocytosis and altered macrophage states may sustain the inflammatory joint microenvironment [14,15,16]. These observations do not establish a direct human semaglutide effect, but they justify examining whether clinical and inflammatory outcomes always move in proportion to weight loss.

The primary objective of this study was therefore to characterize treatment-response heterogeneity in adults with KOA and overweight or obesity treated with semaglutide in routine care. We quantified six-month changes in pain, function, anthropometry, systemic inflammation, glycemia, lipids, dose exposure, and safety; assessed the joint associations of weight loss and achieved dose with improvement; and developed exploratory clinical response phenotypes. We hypothesized that the cohort would contain clinically distinguishable patterns reflecting differences in baseline osteoarthritis severity, inflammatory and metabolic burden, achieved dose, magnitude of weight loss, and concordance or discordance among clinical and biological responses.

## 2. Materials and Methods

### 2.1. Study Design, Setting, and Reporting Framework

We performed a retrospective, observational, self-controlled longitudinal cohort study using de-identified routinely collected data from the Rheumatology Center of Azienda Sanitaria Locale (ASL) Roma 4, San Paolo Hospital, Civitavecchia, Italy. The observation window extended from December 2024 to April 2026. Each patient served as their own comparator across baseline, 3-month, and 6-month assessments after semaglutide initiation. Reporting was informed by the Strengthening the Reporting of Observational Studies in Epidemiology (STROBE) statement and the REporting of studies Conducted using Observational Routinely-collected health Data (RECORD) extension [17,18]. This design permits description of temporal within-patient change but, without an untreated concurrent comparator or randomized exposure, cannot estimate a semaglutide treatment effect or support causal attribution.

### 2.2. Participants and Eligibility

Eligible participants were adults with documented clinical and/or radiographic KOA who received semaglutide for weight management or an obesity-related metabolic indication, had a baseline assessment before treatment initiation, and had at least one post-baseline assessment. Exclusion criteria were incomplete or non-verifiable records, inflammatory arthritis as the dominant knee diagnosis, major knee trauma or surgery close to baseline, total knee replacement before follow-up completion, or initiation of another major weight loss drug or bariatric surgery during the observation period when the record could not be interpreted separately. Data were extracted from clinical notes, laboratory records, prescription records, and follow-up documentation. Identifiers were removed before analysis.

### 2.3. Exposure and Outcomes

Exposure variables included dose-escalation status, maximum weekly dose achieved, final recorded dose, treatment persistence, adverse events, and factors limiting titration. Routine dose levels were 0.25, 0.5, 1.0, 1.7, and 2.4 mg weekly. Achieved dose was treated as an observational exposure because it was influenced by tolerability, adherence, clinical judgment, supply, and patient preference.

Clinical outcomes were visual analog scale (VAS) pain score (0–10; lower scores indicate less pain) and Western Ontario and McMaster Universities Osteoarthritis Index (WOMAC) total score (0–96; lower scores indicate better status) [19]. Anthropometric outcomes were weight and body mass index (BMI). Inflammatory outcomes were C-reactive protein (CRP; mg/L), erythrocyte sedimentation rate (ESR; mm/h), and ferritin (ng/mL). Metabolic and lipid outcomes were glycated hemoglobin (HbA1c; %), low-density lipoprotein cholesterol (LDL-C), high-density lipoprotein cholesterol (HDL-C), and triglycerides (mg/dL). Safety outcomes included treatment persistence, documented abdominal pain, clinically suspected pancreatitis, discontinuation for pancreatic concern, and available amylase, lipase, alanine aminotransferase, and aspartate aminotransferase trajectories.

### 2.4. Statistical Analysis

Continuous variables were summarized using mean and standard deviation, with medians and interquartile ranges where informative; categorical variables were summarized as counts and percentages. Baseline-to-3-month and baseline-to-6-month changes were evaluated using paired *t* tests with 95% confidence intervals (CIs) and Cohen dz. Wilcoxon signed-rank tests and random-intercept mixed models were used for robustness analyses. Percentage weight loss was calculated as 100 × (baseline weight − six-month weight)/baseline weight.

Pearson and Spearman coefficients described unadjusted associations between percentage weight loss and VAS, WOMAC, CRP, ESR, and HbA1c improvement. For each outcome, a hierarchical multivariable linear model with heteroskedasticity-consistent type 3 (HC3) robust standard errors included age, sex, baseline BMI, Kellgren–Lawrence grade, diabetes status, and the baseline value of the outcome, followed by percentage weight loss and maximum achieved dose. Collinearity was assessed using variance inflation factors. An exploratory bootstrapped dose-path decomposition was used for mechanistic orientation but not interpreted as causal mediation because the dose was not randomized and unmeasured confounding could not be excluded. Detailed model specifications, diagnostics, sensitivity analyses, and bootstrap procedures are provided in Supplementary Sections S2–S4. Analyses and figures were implemented in Python version 3.12.13 using NumPy version 2.3.5, pandas version 2.2.3, SciPy version 1.17.0, scikit-learn version 1.8.0, and Matplotlib version 3.10.8 (Python Software Foundation and the respective open-source development teams; https://www.python.org/ and https://pypi.org/; accessed on 20 July 2026).

### 2.5. Exploratory Response Phenotyping

Response phenotyping was intended to describe clinically recognizable patterns rather than to establish validated biological subtypes. K-means clustering was performed on standardized maximum achieved dose, percentage weight loss, BMI decrease, VAS improvement, WOMAC improvement, CRP reduction, ESR reduction, and HbA1c reduction. Candidate solutions from k = 2 to k = 6 were evaluated using silhouette, Calinski–Harabasz, and Davies–Bouldin indices [20,21,22]; cluster size; and repeated 80% subsampling. The two-cluster solution provided the strongest broad statistical partition and separated lower from higher overall multidomain response intensity. Because the clinical objective was to examine heterogeneity within that broad gradient, the five-cluster solution was retained as a secondary, clinically oriented decomposition that distinguished differences in achieved exposure, baseline disease burden, magnitude of weight loss, and concordance or discordance among symptomatic and biological outcomes. This choice was clinical and descriptive, not evidence that k = 5 was statistically optimal. Phenotype labels were assigned only after inspection of baseline and follow-up characteristics. Accordingly, the five clusters should be interpreted as exploratory response patterns within a continuum, not as fixed patient categories or proven mechanistic subtypes. Full selection metrics, stability analyses, and pairwise comparisons are reported in Supplementary Section S5. Because derivation and assessment used the same modest sample, resampling quantified internal stability only and did not constitute independent or external validation.

### 2.6. Missing Data and Bias Considerations

Analyses used complete cases for each outcome pair and no values were imputed. Fasting glucose, fasting insulin or homeostatic model assessment of insulin resistance (HOMA-IR), waist circumference, and standardized knee swelling or imaging measures were unavailable or insufficiently complete. Time-varying physical activity, dietary change, formal physiotherapy or home exercise, and intra-articular corticosteroid or hyaluronic acid injections were not captured consistently. Medication reconciliation was likewise incomplete for oral and topical non-steroidal anti-inflammatory drugs (NSAIDs), paracetamol, opioids, other analgesics, and changes in dose or combination therapy. These omissions prevented adjusted analyses of co-interventions and were treated as sources of residual and time-varying confounding.

### 2.7. Ethics and Data Governance

**Figure 1:**
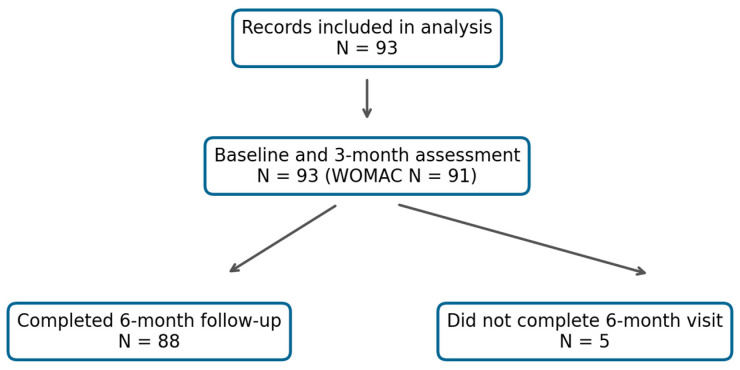
Cohort disposition and analytic sample. WOMAC, Western Ontario and McMaster Universities Osteoarthritis Index.

The study was reviewed and approved by the Comitato Etico Territoriale Lazio Area 3 (protocol Retro-KOA-2026-01; ID 27580 and conducted in accordance with the Declaration of Helsinki. All participants had previously provided written informed consent for the collection and use of their clinical data for research. The analytic dataset was de-identified before analysis (Figure 1).

## 3. Results

### 3.1. Cohort Disposition and Baseline Characteristics

The cohort included 93 adults with KOA and overweight or obesity who initiated semaglutide. Baseline and 3-month data were available for all 93 patients for most outcomes; WOMAC data were available for 91 at 3 months. Eighty-eight patients completed a six-month follow-up. The mean age was 53.9 years, 70 patients (75.3%) were female, and 65 (69.9%) had bilateral KOA. Thirty-eight patients (40.9%) had Kellgren–Lawrence grade 2 and 55 (59.1%) had grade 3 disease. The mean baseline BMI was 35.2 kg/m^2^, VAS pain score was 6.5/10, and WOMAC total score was 61.3/96 (Table 1).

### 3.2. Semaglutide Exposure, Persistence, and Safety Events

Dose escalation was completed in 65 patients (69.9%), delayed in 23 (24.7%), and stopped in 5 (5.4%). The final recorded dose was 2.4 mg in 65 patients, 1.7 mg in 20, and 1.0 mg or less in 8. At follow-up, 88 patients (94.6%) remained on active treatment. One patient had documented abdominal pain; no clinically suspected pancreatitis and no discontinuation for pancreatic concern were recorded (Table 2).

### 3.3. Longitudinal Clinical and Biological Changes

**Figure 2:**
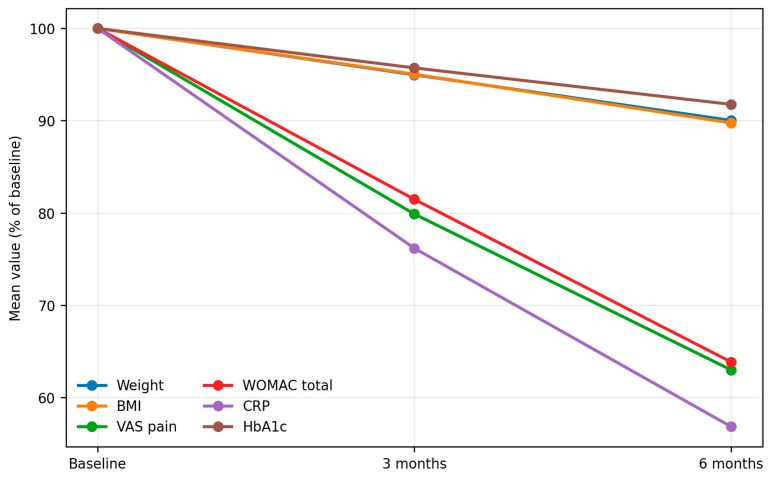
Longitudinal trajectories for selected outcomes normalized to the relevant baseline mean. Lower values indicate improvement for all displayed outcomes. BMI, body mass index; CRP, C-reactive protein; HbA1c, glycated hemoglobin; VAS, visual analog scale; WOMAC, Western Ontario and McMaster Universities Osteoarthritis Index.

Improvement was evident by 3 months and increased by 6 months. At six months, mean weight decreased by 10.88 kg, BMI by 3.87 kg/m^2^, VAS pain score by 2.47 points, and WOMAC total score by 22.50 points. CRP decreased by 2.67 mg/L, ESR by 7.62 mm/h, ferritin by 17.56 ng/mL, and HbA1c by 0.51 percentage points. LDL-C and triglycerides decreased by 17.47 and 33.08 mg/dL, while HDL-C increased by 4.56 mg/dL. All paired comparisons were significant at *p* < 0.001 and were supported by Wilcoxon and mixed-model sensitivity analyses (Table 3; Figure 2; Supplementary Tables S2 and S3).

Clinical relevance was considered in addition to statistical significance. The mean VAS reduction of 2.47 points on a 0–10 scale equals 24.7 points on a 0–100 scale and exceeds the 19.9-point absolute minimal clinically important improvement (MCII) reported for knee osteoarthritis pain. The 22.50-point decrease in WOMAC total corresponds to 36.5% of the baseline mean; this is greater than the 26% relative MCII reported for the WOMAC function subscale, but only as an indirect benchmark because this study used the WOMAC total score, and MCII varies with baseline severity and context [23]. Group means do not establish the proportion of individual responders, which was not prespecified.

### 3.4. Joint Associations of Weight Loss and Achieved Dose

**Figure 3:**
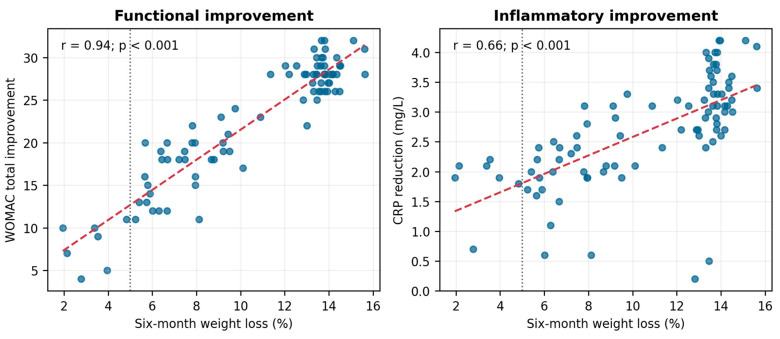
Unadjusted associations of six-month percentage weight loss with WOMAC total score improvement and CRP reduction. The red dashed lines indicate fitted unadjusted linear regressions, and the vertical dotted line denotes 5% weight loss. CRP, C-reactive protein; WOMAC, Western Ontario and McMaster Universities Osteoarthritis Index.

The mean six-month weight loss was 10.56% ± 3.81%; 7 patients lost <5%, 31 lost 5% to <10%, and 50 lost ≥10%. Percentage weight loss correlated strongly with WOMAC (r = 0.94) and VAS score improvement (r = 0.86) and showed a moderate correlation with CRP (r = 0.66), ESR (r = 0.59), and HbA1c reduction (r = 0.59); all *p* < 0.001 (Figure 3). These unadjusted correlations are consistent with, but do not prove, contributions from mechanical unloading and weight-related metabolic change.

In joint multivariable models containing percentage weight loss and maximum achieved dose, weight loss retained an independent association with WOMAC score improvement (adjusted beta 0.848 points per 1% additional weight loss; 95% CI, 0.546–1.150; *p* < 0.001). The weight loss coefficient for CRP reduction was smaller and borderline (0.032 mg/L per 1%; 95% CI, −0.001 to 0.065; *p* = 0.060), while adjusted coefficients for VAS score, ESR, and HbA1c were not statistically significant. The maximum achieved dose retained positive adjusted associations with VAS score, WOMAC score, CRP, ESR, and HbA1c improvement (Table 4). Because dose selection and escalation were not randomized, the dose coefficients are associations rather than causal dose–response effects.

In this observational analysis, weight loss accounted for an important component of functional improvement. Residual dose associations and discordant response patterns were retained as hypothesis-generating signals only; they may reflect adherence, treatment duration, clinical selection, tolerability, co-interventions, or other unmeasured factors and cannot establish additional pharmacologic pathways. Experimental evidence provides biological plausibility for GLP-1 receptor effects relevant to osteoarthritis, but the present data do not test mechanisms. Full hierarchical models, collinearity diagnostics, and bootstrapped path decompositions are provided in Supplementary Tables S4–S6.

### 3.5. Exploratory Clinical Response Phenotypes

**Figure 4:**
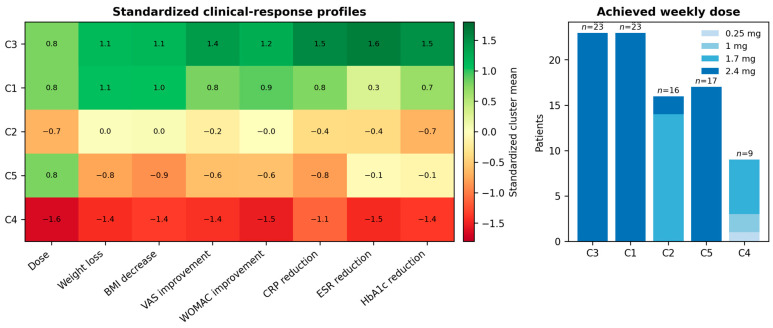
Exploratory five-cluster response phenotypes. The heatmap displays standardized cluster means across exposure and response variables; the adjacent panel shows the underlying distribution of achieved weekly doses. BMI, body mass index; CRP, C-reactive protein; ESR, erythrocyte sedimentation rate; HbA1c, glycated hemoglobin; VAS, visual analog scale; WOMAC, Western Ontario and McMaster Universities Osteoarthritis Index.

All five-cluster findings reported below are secondary, exploratory, and not externally validated. The cluster analysis should be read at two complementary levels. The statistically stronger two-cluster solution captured a broad lower-versus-higher multidomain response gradient. The clinically oriented five-cluster solution then subdivided that gradient into patterns defined by baseline burden, achieved dose, magnitude of weight loss, and whether clinical and biological outcomes changed concordantly. The five clusters are therefore not ranked efficacy classes or immutable biological subtypes; they are descriptive combinations of starting phenotype, treatment exposure, and observed response. The five-cluster model showed lower global separation than the two-cluster model but moderate internal resampling stability (mean adjusted Rand index of 0.736; mean assignment agreement of 84.2%) and was retained to describe clinically meaningful heterogeneity (Figure 4; Table 5 and Table 6). These statistics assess reproducibility within the source dataset, not transportability to new patients. The two-cluster solution remained a robustness sensitivity analysis (Supplementary Tables S7–S9).

C3, the high-inflammatory-burden multidomain responder phenotype (*n* = 23), included patients with the highest baseline pain, WOMAC score, CRP, ESR, and HbA1c burden. All reached a dose of 2.4 mg and lost 13.83% of baseline weight. They achieved the largest pain, functional, inflammatory, and glycemic improvements. C1, the high-dose concordant responder phenotype (*n* = 23), also reached a dose of 2.4 mg and had almost identical weight loss (13.62%), but entered treatment with lower pain, WOMAC, CRP, and ESR than C3 and consequently showed somewhat smaller absolute improvement. Direct comparison confirmed that C3 and C1 had similar weight loss but significantly different baseline inflammatory and symptomatic burden and different response magnitude (Supplementary Table S10). Their separation therefore reflects baseline severity and inflammatory phenotype rather than dose or weight loss differences alone.

C2, the dose-modified intermediate responder phenotype (*n* = 16), was older, had longer KOA duration, and had the highest proportion of Kellgren–Lawrence grade 3 disease. Fourteen patients achieved a dose of 1.7 mg and two achieved 2.4 mg. Weight loss (9.54%) and multidomain improvement were intermediate, suggesting that structural severity, longer disease duration, and modified exposure may constrain response.

C5, the weight-discordant/inflammation-preserved responder phenotype (*n* = 17), consisted entirely of women who reached a dose of 2.4 mg but had lower baseline BMI and milder symptomatic and inflammatory burden. Mean weight loss was 6.49%, yet CRP fell by 1.94 mg/L (46.3% of baseline), ESR by 7.12 mm/h (34.1%), and HbA1c by 0.46 percentage points. Within this restricted cluster, inflammatory reductions were not closely correlated with the magnitude of weight loss. C5 is therefore a hypothesis-generating pattern in this dataset: inflammatory and metabolic changes were relatively preserved despite moderate weight loss. It does not demonstrate weight-independent pharmacology, because the cluster was post hoc, small, and vulnerable to residual confounding and regression to the mean; it identifies a question for prospective mechanistic testing.

C4, the dose-limited graded-response phenotype (*n* = 9), was older and predominantly had grade 3 KOA. Six patients achieved a dose of 1.7 mg, two achieved 1.0 mg, and one achieved 0.25 mg; the mean dose of 1.38 mg is therefore the arithmetic average of these discrete categories rather than a prescribed dose level. Mean weight loss was 3.86%, and clinical improvement was smaller than in the higher-exposure clusters; nevertheless, CRP, ESR, and HbA1c still decreased measurably. This is compatible with a graded observational pattern but cannot be interpreted as a dose–response effect because the achieved dose was selected in routine care and the cluster contained only nine patients.

### 3.6. Safety Laboratory Trajectories

Mean amylase and lipase increased modestly over the follow-up, while alanine and aspartate aminotransferases decreased. No laboratory trajectory was accompanied by clinically suspected pancreatitis in the available records. These descriptive findings are reassuring but cannot estimate uncommon adverse-event risk in a cohort of this size (Supplementary Figure S4 and Table S12).

## 4. Discussion

### 4.1. Principal Findings

This uncontrolled routine-care cohort showed six-month within-patient improvements across body weight, pain, physical function, inflammatory markers, glycemic status, and lipid profile. The central descriptive finding was heterogeneity in within-patient change, organized into candidate patterns defined by baseline disease burden, achieved dose, magnitude of weight loss, and concordance or discordance between clinical and biological improvement. The five-cluster framework described these candidate patterns, while the two-cluster sensitivity analysis and resampling supported only internal structural consistency; neither constitutes external validation.

### 4.2. Clinical and Biological Basis of Response Heterogeneity

The five phenotypes are best interpreted as positions within a multidimensional response space rather than as five mutually exclusive mechanisms. The two-cluster sensitivity solution confirmed a broad gradient of overall response intensity, whereas the five-cluster model explained why patients occupied different positions on that gradient. C3 and C1 both reached a dose of 2.4 mg and lost approximately 13.7% of baseline weight, but C3 began with substantially greater pain, functional impairment, systemic inflammation, and glycemic burden and subsequently showed larger absolute improvement. This difference may partly reflect greater room for improvement and regression to the mean, but it also suggests that baseline inflammatory-metabolic burden is clinically relevant when interpreting response. C2 combined older age, longer KOA duration, a high prevalence of grade 3 radiographic disease, and modified dose exposure; these features were associated with an intermediate multidomain response and may identify patients in whom structural disease and limited exposure constrain symptom recovery.

C5 was the clearest discordant pattern in the source dataset. Its relatively preserved inflammatory and metabolic changes despite moderate weight loss generate a hypothesis for future immunometabolic and synovial studies but do not isolate a weight-independent mechanism. C4 showed a different pattern: lower achieved exposure, modest weight loss, older age, and advanced radiographic disease coincided with smaller clinical changes and measurable biological changes. These observations are compatible with a graded pattern but cannot establish an exposure–response relationship. Descriptively, the clusters reflect mechanical unloading, baseline inflammatory-metabolic burden, structural and pain-related disease severity, and achieved exposure, with probable additional heterogeneity from tolerability, adherence, rehabilitation, diet, analgesic changes, and other unmeasured co-interventions.

### 4.3. Cautious Interpretation of Pathways Beyond Observed Weight Loss

Weight reduction remains a compelling explanation: the strong unadjusted correlations with VAS and WOMAC scores and the adjusted association with function are consistent with reduced joint loading, improved mobility, and downstream metabolic change. Residual dose coefficients and the C5 discordance do not establish independence from weight loss because dose and weight change were collinear and neither was randomized; achieved dose also captured adherence, treatment duration, tolerability, and clinician selection. Unmeasured activity, analgesic changes, diet, injections, and regression to the mean could produce or amplify the observed patterns. Accordingly, these findings are treated as hypotheses about interacting pathways, not as evidence of a direct anti-inflammatory effect independent of weight loss.

Synovial macrophages are biologically plausible candidates in a multi-pathway model. Obesity-associated metabolic dysfunction can promote pro-inflammatory macrophage infiltration in synovium before overt cartilage degradation [14]. In human knee osteoarthritis, synovial macrophage efferocytosis is impaired [15], while transcriptomic profiling identifies heterogeneous macrophage states [16]. Experimental GLP-1 receptor activation has shifted macrophages toward inflammation-resolving phenotypes and reduced local inflammatory mediators [11]. Such effects could influence systemic CRP and ESR through both adipose and joint inflammatory compartments while modifying the local synovial microenvironment.

Chondrocyte, neuroinflammatory, and systemic immunometabolic pathways are also plausible. In experimental models, GLP-1 receptor activation inhibited nuclear factor kappa B (NF-κB) signaling, reduced interleukin-6 (IL-6) and tumor necrosis factor alpha (TNF-α) release, attenuated endoplasmic reticulum stress and apoptosis, and reduced cartilage matrix catabolism [12]. Protein kinase A/cyclic adenosine monophosphate response element-binding protein (PKA/CREB) signaling has likewise been implicated in anti-inflammatory effects in experimental KOA [13]. Reduced synovial cytokine signaling could decrease peripheral nociceptor sensitization and improve movement before structural change occurs, while improved insulin sensitivity and adipose tissue inflammation may reduce the systemic inflammatory input to the joint. The C5 and C4 patterns motivate—but do not test—hypotheses about partial dissociation between observed weight change and biological markers. They are not evidence for a single weight-independent mechanism or a causal dose gradient.

Accordingly, the most defensible interpretation is that the observed response architecture is compatible with broader immunometabolic effects of GLP-1 receptor agonism, while remaining insufficient to prove that such effects are independent of weight loss. CRP and ESR are non-specific systemic markers; no synovial fluid, imaging, cytokine, tissue, body composition, or physical activity measurements were available; pain improvement can reflect multiple non-inflammatory processes. Future prospective studies should combine standardized titration and co-intervention capture with ultrasound or magnetic resonance imaging (MRI) of synovitis, adipokines, cytokines, macrophage-related biomarkers, pain-sensitization measures, and repeated mediator assessment. Such designs are required to determine whether immunometabolic and synovial pathways contribute materially to disease activity beyond the reduction in mechanical load. Such studies also require a concurrent comparator and comprehensive time-varying capture of medications and other co-interventions.

### 4.4. Relationship to STEP 9 and Existing Clinical Evidence

STEP 9 provides the principal randomized semaglutide evidence in obesity-related KOA: 407 participants at 61 sites received semaglutide at a dose of 2.4 mg or placebo plus lifestyle counseling for 68 weeks. The mean weight change was −13.7% versus −3.2%, the WOMAC pain score change was −41.7 versus −27.5 points, and the WOMAC physical-function score change was −41.5 versus −26.7 points [8]. The present cohort showed a mean weight loss of 10.56% at six months, a 2.47/10 VAS score reduction, and a 22.50/96 WOMAC total score reduction. These values are directionally concordant, but direct numerical comparison is inappropriate because the outcome scales, follow-up duration, baseline case mix, co-interventions, and designs differed. Unlike STEP 9, this study has no control group and cannot estimate improvement attributable to semaglutide above concurrent care, expectancy, or temporal effects.

Semaglutide-specific real-world evidence in KOA remains sparse. A 2025 systematic review found only four human GLP-1RA studies and emphasized heterogeneity in study design and outcomes [9]. The Shanghai Osteoarthritis Cohort associated class-level GLP-1RA use in patients with type 2 diabetes with lower WOMAC scores and knee surgery incidence, with benefit substantially mediated by weight change [10]. That cohort was not semaglutide-specific and differed in population and baseline BMI. Thus, the present findings extend descriptive routine-care evidence but should be viewed as complementary to, not confirmatory of, randomized evidence.

The Conforti studies address a different therapeutic strategy—fixed-dose tramadol/paracetamol for symptomatic analgesia—but are relevant to the clinical context [4,5]. They highlight the value of pragmatic follow-up and rapid pain control in routine KOA care. Semaglutide should not be considered a replacement for individualized analgesia or rehabilitation; its potential contribution is broader, combining obesity treatment with possible downstream effects on function, inflammation, and cardiometabolic risk.

### 4.5. Clinical and Research Implications

Clinically, the phenotype framework suggests that follow-up should assess more than kilograms lost. Patients with high inflammatory and symptomatic burden may show large multidomain gains when target exposure is achieved. Patients with long-standing grade 3 disease may need intensified rehabilitation and structural reassessment even when metabolic markers improve. Patients who lose weight without proportional symptom relief should be evaluated for malalignment, meniscal disease, central sensitization, sleep disturbance, mood, or inadequate exercise progression. Conversely, biological improvement with modest weight loss should prompt continued monitoring rather than immediate classification as treatment failure.

The candidate phenotypes may inform future stratification research by prioritizing baseline symptoms and inflammatory burden, radiographic severity, early weight trajectory, and dose tolerance as prespecified predictors. They should not guide individual treatment selection at present; any prediction rule would require locked definitions, calibration and discrimination testing, and independent prospective validation.

WOMAC and VAS scores are patient-reported and should be complemented in future studies by objective performance measures such as the 6 min walk, timed chair rise, and instrumented gait. Wearable inertial sensors have detected pathological gait features in lumbosacral discopathy [24]; although that condition differs from KOA, the study illustrates how repeatable kinematic data could complement the WOMAC score and reduce reliance on a single measurement modality.

Prospective, controlled, multicenter studies should prespecify phenotype variables and analysis plans; standardize titration and co-interventions; capture time-varying medications, diet, activity, physiotherapy, and injections; measure body composition and objective function; and incorporate ultrasound or MRI synovitis, joint effusion, adipokines, cytokines, macrophage-related biomarkers, and pain-sensitization measures. Repeated measurements would permit appropriately designed mediation analyses. A candidate solution should be derived in one cohort and tested in an independent cohort with preregistered assignment rules. No clinical implementation is warranted until reproducibility, transportability, and incremental predictive value have been demonstrated.

### 4.6. Strengths and Limitations

Strengths include the longitudinal within-patient design; integration of clinical, functional, anthropometric, inflammatory, metabolic, lipid, exposure, and safety domains; explicit joint modeling of weight loss and achieved dose; reporting of robust and mixed-model sensitivity analyses; and transparent evaluation of both parsimonious and clinically granular cluster solutions. The revised phenotyping analysis directly describes who the patients were at baseline and what they achieved during follow-up.

Limitations include retrospective selection and information bias, absence of a concurrent comparator, residual confounding, and regression to the mean. Consequently, within-patient change cannot be attributed to semaglutide, and the joint models cannot establish causal or weight-independent effects. Achieved dose was endogenous and cannot be separated from adherence, treatment duration, tolerability, access, or clinician selection. The sample was modest for multivariable modeling and small within individual phenotypes. The five-cluster model had lower global separation than the two-cluster solution and moderate rather than perfect stability, particularly at the boundary between C1 and C3. Because the same 88 participants were used for derivation and stability assessment, resampling indicates internal reproducibility only, not external validity. No local synovial, imaging, cytokine, body composition, objective gait, or structural outcomes were available. The results therefore identify hypotheses and candidate response patterns, not causal mechanisms or validated biological subtypes.

Medication exposure was not captured comprehensively. Changes in oral or topical NSAIDs, paracetamol, opioids, other analgesics, and medication combinations could influence pain, activity, laboratory markers, and safety signals. Large electronic health record (EHR) analyses demonstrate that concomitant NSAID combinations can be examined systematically for interaction-related safety outcomes such as drug-induced liver injury [25], underscoring the need for time-varying medication reconciliation. Diet, physical activity, formal physiotherapy, home exercise, and intra-articular corticosteroid or hyaluronic acid injections were likewise not standardized or consistently recorded. These omissions leave confounding by indication and co-intervention unresolved.

Generalizability is also limited by the single-center Italian setting and the cohort composition (75.3% women, overweight or obesity, and Kellgren–Lawrence grade 2 or 3 disease). Findings may not transport to men; other racial or ethnic and geographic populations; people with normal BMI, grade 1 or 4 disease, or prior arthroplasty; or health systems with different prescribing, access, rehabilitation, and dose-escalation practices.

## 5. Conclusions

In adults with KOA and overweight or obesity treated with semaglutide in routine care, six-month improvements were observed in weight, pain, function, systemic inflammatory markers, glycemic status, and lipids. The uncontrolled design does not establish that semaglutide caused these changes. Response was heterogeneous, and exploratory clustering described five candidate patterns nested within a broader response-intensity gradient; these are hypothesis-generating patterns, not validated patient subtypes. Weight loss was associated with functional improvement, while residual dose and biomarker patterns may reflect unmeasured confounding and cannot establish non-weight pharmacologic pathways. Prospective controlled, multicenter studies with concurrent comparators, comprehensive co-intervention capture, objective function, local joint biomarkers, repeated mediator measurements, and independent phenotype validation are required before causal claims or clinical stratification.

## Figures and Tables

**Table 1:** Baseline demographic, osteoarthritis, anthropometric, inflammatory, and metabolic characteristics.

Characteristic	Value	Additional Descriptor
Total cohort, *n*	93	100.0%
Age, years	53.9 ± 9.3	Median 54.0
Female sex, *n* (%)	70 (75.3)	
Bilateral KOA, *n* (%)	65 (69.9)	
Kellgren–Lawrence grade 2, *n* (%)	38 (40.9)	
Kellgren–Lawrence grade 3, *n* (%)	55 (59.1)	
Weight, kg	97.1 ± 18.3	Median 98.2
BMI, kg/m^2^	35.2 ± 4.7	Median 36.0
KOA duration, years	5.4 ± 2.4	Median 5.0
VAS pain score, 0–10	6.5 ± 1.1	Median 7.0
WOMAC total score, 0–96	61.3 ± 7.3	Median 63.0
CRP, mg/L	6.0 ± 1.8	Median 6.1
ESR, mm/h	25.3 ± 4.5	Median 26.0
Ferritin, ng/mL	155.1 ± 44.3	Median 149.0
HbA1c, %	6.07 ± 0.39	Median 6.00

Values are shown as mean ± SD unless otherwise stated. BMI, body mass index; CRP, C-reactive protein; ESR, erythrocyte sedimentation rate; KOA, knee osteoarthritis; VAS, visual analog scale; WOMAC, Western Ontario and McMaster Universities Osteoarthritis Index; SD, standard deviation.

**Table 2:** Semaglutide dose escalation, exposure, persistence, and selected safety events.

Exposure or Safety Variable	*n*	%
Completed escalation	65	69.9
Delayed escalation	23	24.7
Stopped escalation	5	5.4
Final dose 2.4 mg	65	69.9
Final dose 1.7 mg	20	21.5
Final dose ≤ 1.0 mg	8	8.6
Active treatment at follow-up	88	94.6
Abdominal pain recorded	1	1.1
Clinically suspected pancreatitis	0	0.0
Discontinued for pancreatic concern	0	0.0

**Table 3:** Six-month changes from baseline. CI, confidence interval; SD, standard deviation.

Outcome	*n*	Baseline Mean ± SD	6-Month Mean ± SD	Mean Change (95% CI)	*p*
Weight, kg	88	98.32 ± 17.99	87.44 ± 13.56	−10.88 (−11.97 to −9.79)	<0.001
BMI, kg/m^2^	88	35.48 ± 4.68	31.61 ± 3.36	−3.87 (−4.23 to −3.52)	<0.001
VAS pain score, 0–10	88	6.57 ± 1.14	4.10 ± 0.66	−2.47 (−2.66 to −2.27)	<0.001
WOMAC total score, 0–96	88	61.60 ± 7.37	39.10 ± 5.53	−22.50 (−24.02 to −20.98)	<0.001
CRP, mg/L	88	6.08 ± 1.78	3.41 ± 1.07	−2.67 (−2.85 to −2.48)	<0.001
ESR, mm/h	88	25.36 ± 4.45	17.74 ± 3.72	−7.62 (−8.03 to −7.22)	<0.001
Ferritin, ng/mL	83	156.18 ± 45.91	138.61 ± 42.72	−17.56 (−18.96 to −16.17)	<0.001
HbA1c, %	88	6.08 ± 0.39	5.57 ± 0.31	−0.51 (−0.55 to −0.47)	<0.001
LDL-C, mg/dL	88	144.27 ± 13.17	126.81 ± 13.76	−17.47 (−18.36 to −16.57)	<0.001
Triglycerides, mg/dL	88	172.02 ± 31.18	138.94 ± 23.03	−33.08 (−35.41 to −30.74)	<0.001
HDL-C, mg/dL	88	44.27 ± 4.74	48.83 ± 4.12	+4.56 (+4.29 to +4.82)	<0.001

**Table 4:** Correlations and joint multivariable models for six-month improvement.

Outcome	Pearson r	Weight Loss Beta (95% CI)	*p*	Dose Beta (95% CI)	*p*	Model R^2^
VAS score improvement	0.86	−0.005 (−0.059 to 0.048)	0.844	0.951 (0.568 to 1.334)	<0.001	0.88
WOMAC score improvement	0.94	0.848 (0.546 to 1.150)	<0.001	5.050 (3.320 to 6.780)	<0.001	0.94
CRP reduction	0.66	0.032 (−0.001 to 0.065)	0.060	0.553 (0.202 to 0.904)	0.002	0.92
ESR reduction	0.59	0.066 (−0.080 to 0.211)	0.376	1.707 (0.363 to 3.051)	0.013	0.69
HbA1c reduction	0.59	0.012 (−0.005 to 0.029)	0.173	0.154 (0.071 to 0.237)	<0.001	0.69

Models adjusted for age, sex, baseline BMI, Kellgren–Lawrence grade, diabetes status, the outcome-specific baseline value, percentage weight loss, and maximum achieved dose. HC3 robust standard errors were used. Weight loss beta is per 1% additional weight loss; dose beta is per 1 mg higher achieved weekly dose. BMI, body mass index; CI, confidence interval; CRP, C-reactive protein; ESR, erythrocyte sedimentation rate; HbA1c, glycated hemoglobin; HC3, heteroskedasticity-consistent type 3; VAS, visual analog scale; WOMAC, Western Ontario and McMaster Universities Osteoarthritis Index.

**Table 5:** Baseline and exposure characteristics of the five exploratory response phenotypes.

Phenotype	*n*	Age	Female *n* (%)	KL3 *n* (%)	BMI	VAS	WOMAC	CRP	ESR	Achieved Dose Distribution
C3 High-inflammatory-burden multidomain	23	51.4 ± 7.7	11 (47.8)	14 (60.9)	38.54	7.61	68.26	7.45	29.00	2.4 mg: 23
C1 High-dose concordant	23	48.5 ± 7.8	17 (73.9)	8 (34.8)	37.83	7.00	63.13	6.54	24.74	2.4 mg: 23
C2 Dose-modified intermediate	16	61.9 ± 9.0	12 (75.0)	14 (87.5)	36.86	6.94	65.50	6.18	27.31	1.7 mg: 14, 2.4 mg: 2
C5 Weight-discordant/inflammation-preserved	17	51.6 ± 7.7	17 (100.0)	8 (47.1)	29.80	5.00	51.29	4.18	20.88	2.4 mg: 17
C4 Dose-limited graded response	9	60.1 ± 6.9	8 (88.9)	7 (77.8)	29.94	5.11	53.22	4.78	22.67	0.25 mg: 1, 1.0 mg: 2, 1.7 mg: 6

Values are mean ± SD where shown. CRP in mg/L; ESR in mm/h. KL3, Kellgren–Lawrence grade 3; BMI, body mass index; SD, standard deviation; VAS, visual analog scale; WOMAC, Western Ontario and McMaster Universities Osteoarthritis Index.

**Table 6:** Six-month response characteristics of the five exploratory phenotypes.

Phenotype	Mean Dose (mg)	Weight Loss (%)	VAS Improvement	WOMAC Improvement	CRP Reduction	ESR Reduction	HbA1c Reduction
C3 High-inflammatory-burden multidomain	2.40	13.83	3.43	29.48	3.55	9.78	0.67
C1 High-dose concordant	2.40	13.62	2.91	27.04	3.00	7.65	0.56
C2 Dose-modified intermediate	1.79	9.54	2.06	20.31	2.23	6.56	0.38
C5 Weight-discordant/inflammation-preserved	2.40	6.49	1.71	16.06	1.94	7.12	0.46
C4 Dose-limited graded response	1.38	3.86	1.00	9.11	1.72	4.89	0.29

CRP reduction is shown in mg/L; ESR reduction in mm/h; and HbA1c reduction in percentage points. Cluster means are descriptive and should not be interpreted as randomized dose effects. CRP, C-reactive protein; ESR, erythrocyte sedimentation rate; HbA1c, glycated hemoglobin; VAS, visual analog scale; WOMAC, Western Ontario and McMaster Universities Osteoarthritis Index.

## Data Availability

The de-identified analytic dataset may be made available by the corresponding author upon reasonable request and subject to institutional governance approval.

## Supplementary Materials

The following supporting information can be downloaded at https://www.mdpi.com/article/10.3390/jcm15155876/s1.

## References

[1] Bannuru R.R., Osani M.C., Vaysbrot E.E., Arden N.K., Bennell K., Bierma-Zeinstra S.M.A., Kraus V.B., Lohmander L.S., Abbott J.H., Bhandari M. (2019). OARSI guidelines for the non-surgical management of knee, hip, and polyarticular osteoarthritis. Osteoarthr. Cartil..

[2] Moseng T., Vliet Vlieland T.P.M., Battista S., Beckwée D., Boyadzhieva V., Conaghan P.G., Costa D., Doherty M., Finney A.G., Georgiev T. (2024). EULAR recommendations for the non-pharmacological core management of hip and knee osteoarthritis: 2023 update. Ann. Rheum. Dis..

[3] Messier S.P., Beavers D.P., Queen K., Mihalko S.L., Miller G.D., Losina E., Katz J.N., Loeser R.F., DeVita P., Hunter D.J. (2022). Effect of diet and exercise on knee pain in patients with osteoarthritis and overweight or obesity: A randomized clinical trial. JAMA.

[4] Conforti A., Ruggiero C., Palombi N., Messina F., Bonifacio M., Lucchetti L., Ruggiero M., La Cava G., Piazza R., Mangrella M. (2025). Evaluation of tramadol/paracetamol 75 mg/650 mg combination therapy for early-stage knee osteoarthritis: A retrospective observational study. Reumatismo.

[5] Cipolloni V., Bonifacio M., Musorrofiti M.G., Saggini R., Caldarola A., Grossi G., Piazza R., Mangrella M., Trastulli D., Conforti A. (2026). Short- and Mid-Term Outcomes of Fixed-Dose Tramadol/Paracetamol in Early-Stage Symptomatic Knee Osteoarthritis: A Single-Center Retrospective Observational Extension Study. Life.

[6] Wilding J.P.H., Batterham R.L., Calanna S., Davies M., Van Gaal L.F., Lingvay I., McGowan B.M., Rosenstock J., Tran M.T., Wadden T.A. (2021). Once-weekly semaglutide in adults with overweight or obesity. N. Engl. J. Med..

[7] Verma S., Bhatta M., Davies M., Deanfield J.E., Garvey W.T., Jensen C., Kandler K., Kushner R.F., Rubino D.M., Kosiborod M.N. (2023). Effects of once-weekly semaglutide 2.4 mg on C-reactive protein in adults with overweight or obesity (STEP 1, 2, and 3): Exploratory analyses of three randomized, double-blind, placebo-controlled phase 3 trials. EClinicalMedicine.

[8] Bliddal H., Bays H., Czernichow S., Udden Hemmingsson J., Hjelmesæth J., Hoffmann Morville T., STEP 9 Study Group (2024). Once-weekly semaglutide in persons with obesity and knee osteoarthritis. N. Engl. J. Med..

[9] Cheng J., Solomon T., Estee M., Cicuttini F.M., Lim Y.Z. (2025). Effect of glucagon-like peptide-1 receptor agonists in osteoarthritis: A systematic review of pre-clinical and human studies. Osteoarthr. Cartil. Open.

[10] Zhu H., Zhou L., Wang Q., Cai Q., Yang F., Jin H., Chen Y., Song Y., Zhang C. (2023). Glucagon-like peptide-1 receptor agonists as a disease-modifying therapy for knee osteoarthritis mediated by weight loss: Findings from the Shanghai Osteoarthritis Cohort. Ann. Rheum. Dis..

[11] Meurot C., Martin C., Sudre L., Breton J., Bougault C., Rattenbach R., Bismuth K., Jacques C., Berenbaum F. (2022). Liraglutide, a glucagon-like peptide-1 receptor agonist, exerts analgesic, anti-inflammatory and anti-degradative actions in osteoarthritis. Sci. Rep..

[12] Chen J., Xie J.J., Shi K.S., Gu Y.T., Wu C.C., Xuan J., Ren Y., Chen L., Wu Y.S., Zhang X.L. (2018). Glucagon-like peptide-1 receptor regulates endoplasmic reticulum stress-induced apoptosis and the associated inflammatory response in chondrocytes and the progression of osteoarthritis in rat. Cell Death Dis..

[13] Que Q., Guo X., Zhan L., Chen S., Zhang Z., Ni X., Ye B., Wan S. (2019). The GLP-1 agonist liraglutide ameliorates inflammation through activation of the PKA/CREB pathway in a rat model of knee osteoarthritis. J. Inflamm..

[14] Sun A.R., Panchal S.K., Friis T., Sekar S., Crawford R., Brown L., Xiao Y., Prasadam I. (2017). Obesity-associated metabolic syndrome spontaneously induces infiltration of pro-inflammatory macrophages in synovium and promotes osteoarthritis. PLoS ONE.

[15] Del Sordo L., Blackler G.B., Philpott H.T., Riviere J., Gunaratnam L., Heit B., Appleton C.T. (2023). Impaired efferocytosis by synovial macrophages in patients with knee osteoarthritis. Arthritis Rheumatol..

[16] Wang C., De Francesco R., Lamers L.A., Rinzema S., Frölich S., van Lent P.L.E.M., Logie C., van den Bosch M.H.J. (2025). Transcriptomic profiling of osteoarthritis synovial macrophages reveals a tolerized phenotype compounded by a weak corticosteroid response. Rheumatology.

[17] von Elm E., Altman D.G., Egger M., Pocock S.J., Gøtzsche P.C., Vandenbroucke J.P. (2007). The Strengthening the Reporting of Observational Studies in Epidemiology (STROBE) statement: Guidelines for reporting observational studies. PLoS Med..

[18] Benchimol E.I., Smeeth L., Guttmann A., Harron K., Moher D., Petersen I., Sørensen H.T., von Elm E., Langan S.M. (2015). The REporting of studies Conducted using Observational Routinely-collected health Data (RECORD) statement. PLoS Med..

[19] Bellamy N., Buchanan W.W., Goldsmith C.H., Campbell J., Stitt L.W. (1988). Validation study of WOMAC: A health status instrument for measuring clinically important patient-relevant outcomes to antirheumatic drug therapy in patients with osteoarthritis of the hip or knee. J. Rheumatol..

[20] Rousseeuw P.J. (1987). Silhouettes: A graphical aid to the interpretation and validation of cluster analysis. J. Comput. Appl. Math..

[21] Calinski T., Harabasz J. (1974). A dendrite method for cluster analysis. Commun. Stat..

[22] Davies D.L., Bouldin D.W. (1979). A cluster separation measure. IEEE Trans. Pattern Anal. Mach. Intell..

[23] Tubach F., Ravaud P., Baron G., Falissard B., Logeart I., Bellamy N., Bombardier C., Felson D., Hochberg M., van der Heijde D. (2005). Evaluation of clinically relevant changes in patient reported outcomes in knee and hip osteoarthritis: The minimal clinically important improvement. Ann. Rheum. Dis..

[24] Głowiński S., Łosiński K., Kowiański P., Waśkow M., Bryndal A., Grochulska A. (2020). Inertial sensors as a tool for diagnosing discopathy lumbosacral pathologic gait: A preliminary research. Diagnostics.

[25] Datta A., Flynn N.R., Barnette D.A., Woeltje K.F., Miller G.P., Swamidass S.J. (2021). Machine learning liver-injuring drug interactions with non-steroidal anti-inflammatory drugs (NSAIDs) from a retrospective electronic health record (EHR) cohort. PLoS Comput. Biol..

