# Supplementary material for: Beyond the Scale: Changes in Pain, Physical Function (WOMAC), and Low-Grade Inflammation Following Semaglutide Treatment in Patients with Knee Osteoarthritis—Results from a Six-Month Real-World Cohort
Source: J Clin Med. 2026 Jul 27;15(15):5876. doi: 10.3390/jcm15155876 (PMC13466628; doi:10.3390/jcm15155876)
Supplement: Supplementary file 1 [file jcm-15-05876-s001.zip › jcm-4419166-supplementary.pdf]

Supplementary Materials

Semaglutide Treatment in Patients with Knee Osteoarthritis and Overweight or Obesity: A Six-Month Retrospective Cohort with Exploratory Response Phenotyping

S1. Purpose and Organization of the Supplementary Analyses

These supplementary analyses were designed to document the reproducibility, robustness, and limitations of the main findings without obscuring their clinical interpretation. Sections S2 and S3 verify the longitudinal changes using alternative distributional and repeated-measures approaches. Section S4 evaluates whether weight loss and achieved dose provide separable statistical information and documents the exploratory path decomposition. Section S5 explains why the clinically granular five-cluster solution was retained in the main manuscript while the more parsimonious two-cluster solution was used as a robustness sensitivity analysis. Section S6 provides safety laboratory details. None of these analyses converts the observational design into a causal experiment.

S2. Data Completeness and Longitudinal Robustness

Table S1. Outcome availability by time point.

Outcome	Baseline n	3-month n	6-month n
Weight/BMI/VAS/CRP/HbA1c/lipids	93	93	88
WOMAC total	93	91	88
ESR	93	88	88
Ferritin	93	93	83

Table S2. Paired changes, distribution-robust sensitivity tests, and standardized effects.

Outcome	Time	Mean change (95% CI)	Paired/Wilcoxon p	|Cohen dz|
Weight	3 m	-4.90 (-5.42,-4.37)	<0.001	1.92
Weight	6 m	-10.88 (-11.97,-9.79)	<0.001	2.12
BMI	3 m	-1.74 (-1.92,-1.57)	<0.001	2.06
BMI	6 m	-3.87 (-4.23,-3.52)	<0.001	2.31
VAS	3 m	-1.30 (-1.45,-1.15)	<0.001	1.81
VAS	6 m	-2.47 (-2.66,-2.27)	<0.001	2.64
WOMAC	3 m	-11.35 (-12.31,-10.39)	<0.001	2.46
WOMAC	6 m	-22.50 (-24.02,-20.98)	<0.001	3.13
CRP	3 m	-1.43 (-1.54,-1.32)	<0.001	2.77
CRP	6 m	-2.67 (-2.85,-2.48)	<0.001	3.00
ESR	3 m	-3.90 (-4.13,-3.67)	<0.001	3.56
ESR	6 m	-7.62 (-8.03,-7.22)	<0.001	4.02
Ferritin	3 m	-7.87 (-8.48,-7.26)	<0.001	2.64
Ferritin	6 m	-17.56 (-18.96,-16.17)	<0.001	2.75
HbA1c	3 m	-0.25 (-0.27,-0.23)	<0.001	2.60
HbA1c	6 m	-0.51 (-0.55,-0.47)	<0.001	2.81
LDL-C	3 m	-7.54 (-7.92,-7.15)	<0.001	4.02
LDL-C	6 m	-17.47 (-18.36,-16.57)	<0.001	4.12
Triglycerides	3 m	-15.47 (-16.75,-14.20)	<0.001	2.49
Triglycerides	6 m	-33.08 (-35.41,-30.74)	<0.001	3.00
HDL-C	3 m	+1.94 (+1.78,+2.09)	<0.001	2.64
HDL-C	6 m	+4.56 (+4.29,+4.82)	<0.001	3.65

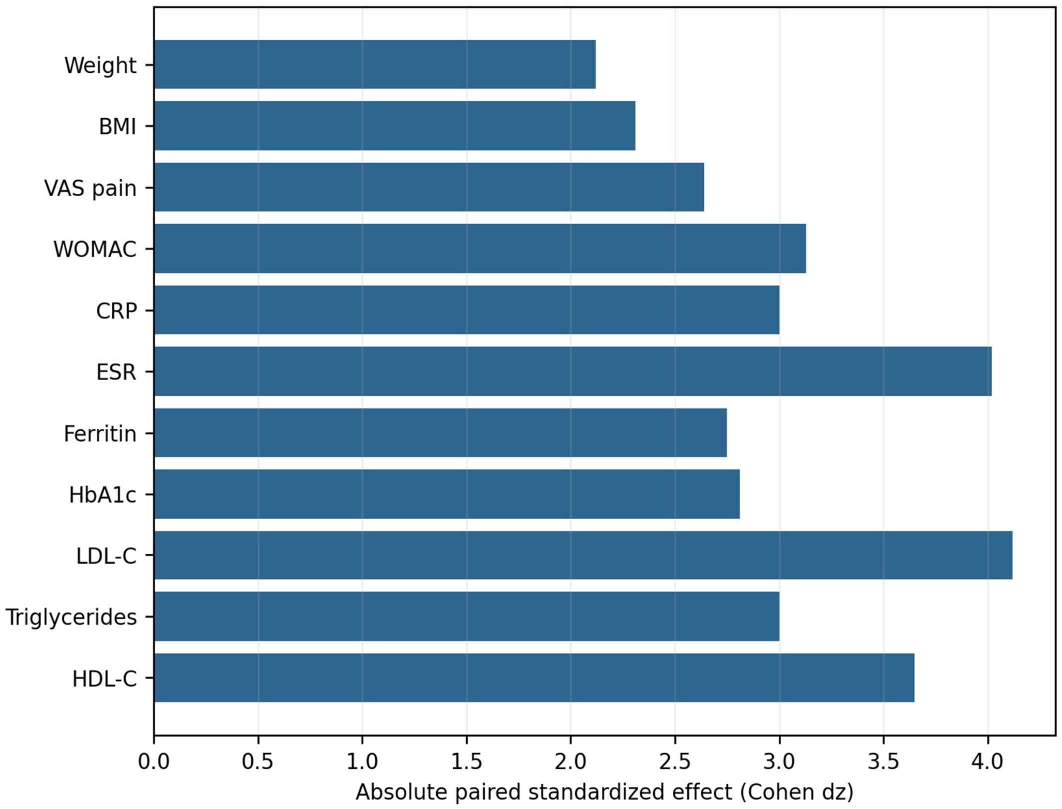

Figure S1. Absolute paired standardized effect sizes at six months. Positive bar lengths indicate larger improvement magnitude; directionality was harmonized for display.

Table S3. Random-intercept mixed-model time coefficients (reference: baseline).

Outcome	Time	Coefficient	95% CI	p
Weight, kg	3 m	-4.898	-5.638 to -4.158	<0.001
Weight, kg	6 m	-10.763	-11.518 to -10.007	<0.001
BMI, kg/m²	3 m	-1.743	-1.985 to -1.501	<0.001
BMI, kg/m²	6 m	-3.828	-4.076 to -3.581	<0.001
VAS	3 m	-1.301	-1.452 to -1.150	<0.001
VAS	6 m	-2.423	-2.577 to -2.270	<0.001
WOMAC	3 m	-11.312	-12.395 to -10.229	<0.001
WOMAC	6 m	-22.229	-23.325 to -21.133	<0.001
CRP, mg/L	3 m	-1.431	-1.560 to -1.302	<0.001
CRP, mg/L	6 m	-2.645	-2.777 to -2.513	<0.001
ESR, mm/h	3 m	-3.853	-4.143 to -3.563	<0.001

Outcome	Time	Coefficient	95% CI	p
ESR, mm/h	6 m	-7.584	-7.874 to -7.294	<0.001
Ferritin, ng/mL	3 m	-7.869	-8.777 to -6.961	<0.001
Ferritin, ng/mL	6 m	-17.473	-18.421 to -16.524	<0.001
HbA1c, %	3 m	-0.253	-0.280 to -0.226	<0.001
HbA1c, %	6 m	-0.505	-0.532 to -0.477	<0.001
LDL-C, mg/dL	3 m	-7.538	-8.160 to -6.916	<0.001
LDL-C, mg/dL	6 m	-17.378	-18.013 to -16.743	<0.001
Triglycerides, mg/dL	3 m	-15.473	-17.075 to -13.871	<0.001
Triglycerides, mg/dL	6 m	-32.801	-34.437 to -31.166	<0.001
HDL-C, mg/dL	3 m	1.935	1.750 to 2.121	<0.001
HDL-C, mg/dL	6 m	4.528	4.339 to 4.717	<0.001

Models included a patient-specific random intercept and categorical time. The mixed-model estimates closely reproduced the paired analyses, supporting robustness to incomplete follow-up under model assumptions.

S3. Weight Loss, Dose Exposure, and Model Diagnostics

The purpose of these analyses was not to label a residual dose coefficient as a direct drug effect. Instead, the models assessed whether the observed variation could be represented by weight loss alone or whether dose exposure and baseline characteristics retained additional statistical information. Because dose and weight loss were interrelated, coefficient attenuation and p values were interpreted together with confidence intervals, partial R^2, and variance inflation factors.

Table S4. Full joint regression estimates (HC3 robust standard errors).

Outcome	Weight-loss beta (95% CI)	p	Dose beta (95% CI)	p	R^2
VAS	-0.005 (-0.059,0.048)	0.844	0.951 (0.568,1.334)	<0.001	0.88
WOMAC	0.848 (0.546,1.150)	<0.001	5.050 (3.320,6.780)	<0.001	0.94
CRP	0.032 (-0.001,0.065)	0.060	0.553 (0.202,0.904)	0.002	0.92
ESR	0.066 (-0.080,0.211)	0.376	1.707 (0.363,3.051)	0.013	0.69
HbA1c	0.012 (-0.005,0.029)	0.173	0.154 (0.071,0.237)	<0.001	0.69

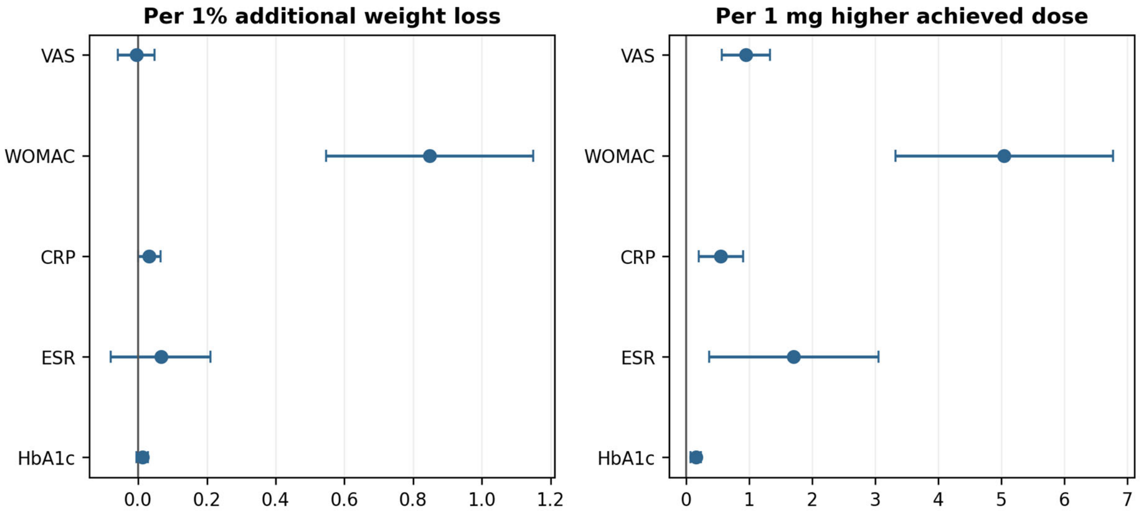

Figure S2. Adjusted associations of percentage weight loss and maximum achieved dose with outcome improvement. Units differ across outcomes.

Table S5. Collinearity and interpretive safeguards.

Diagnostic/issue	Finding	Interpretive consequence
Weight-loss VIF	Approximately 5.3-8.1 across full models	Moderate collinearity can widen CIs and destabilize separate weight and dose coefficients.
Dose assignment	Not randomized	Dose coefficients may reflect adherence, duration, tolerability, clinical selection, and unmeasured factors.
Outcome power	n=88; multiple covariates	Non-significance is not evidence that weight loss is unimportant.
Temporal structure	Dose and weight accumulated over follow-up	Simultaneous six-month variables do not establish mediator ordering.

S4. Exploratory Dose-Path Decomposition

The path decomposition was included to organize hypotheses for future prospective research. It quantified the descriptive product of the dose-to-weight association and the weight-to-outcome association conditional on dose and covariates, and contrasted that product with the remaining dose-outcome association. The analysis does not satisfy the assumptions for causal mediation.

Table S6. Exploratory dose-path coefficients (1200 bootstrap samples).

Outcome	Dose→weight path a	Dose-through-weight product (95% CI)	Residual dose association (95% CI)	Total dose association (95% CI)
VAS	3.89	-0.021 (-0.210,0.176)	0.951 (0.594,1.333)	0.932 (0.662,1.218)
WOMAC	3.89	3.303 (1.979,4.690)	5.050 (3.349,6.793)	8.051 (6.519,9.851)
CRP	3.89	0.124 (-0.016,0.250)	0.553 (0.227,0.832)	0.679 (0.381,0.910)
ESR	3.89	0.255 (-0.338,0.810)	1.707 (0.954,3.189)	1.959 (1.319,3.319)
HbA1c	3.89	0.047 (-0.008,0.113)	0.154 (0.078,0.237)	0.200 (0.119,0.267)

All coefficients are per 1 mg higher achieved weekly dose. These are descriptive path coefficients, not natural direct or indirect effects.

S5. Cluster Selection, Validation, and Detailed Phenotype Analyses

The objective of the cluster analysis was clinical phenotyping, not merely optimization of an internal index. The two-cluster solution provided the strongest global separation and is reported as a parsimonious sensitivity result. The five-cluster solution was retained in the main manuscript because it separated high-inflammatory-burden responders, high-dose concordant responders, dose-modified intermediate responders, weight-discordant/inflammation-preserved

responders, and dose-limited modest responders. The trade-off is lower global separation and only moderate stability; this is stated explicitly.

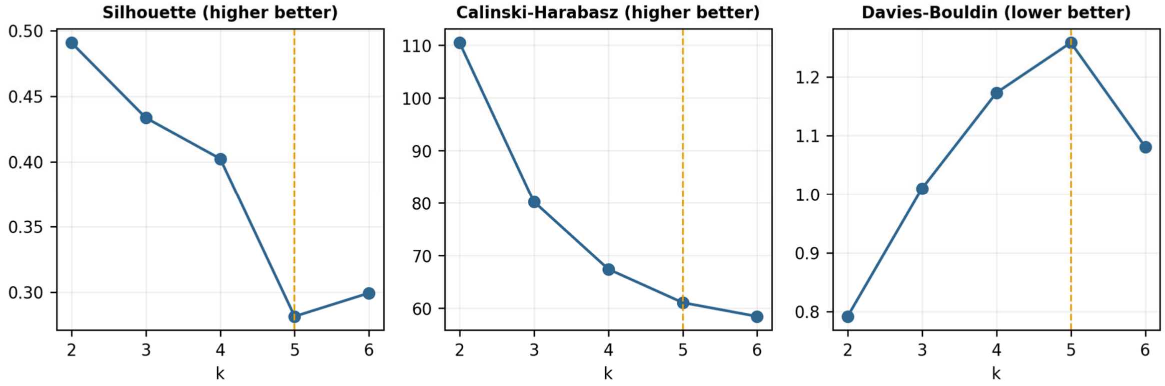

Figure S3. Internal validation metrics for candidate K-means solutions. The dashed line marks the clinically oriented five-cluster phenotyping model.

Table S7. Candidate cluster solutions.

k	Silhouette	Calinski-Harabasz	Davies-Bouldin	Cluster sizes
2	0.491	110.5	0.792	40, 48
3	0.433	80.2	1.010	8, 32, 48
4	0.402	67.4	1.173	8, 17, 19, 44
5	0.282	61.1	1.258	9, 16, 17, 23, 23
6	0.299	58.5	1.081	2, 9, 14, 17, 23, 23

Table S8. Five-cluster resampling stability.

Metric	Estimate
Mean adjusted Rand index	0.736
Median adjusted Rand index	0.688
Empirical 2.5th-97.5th percentile ARI	0.526-0.976
Mean participant assignment agreement	84.2%
Empirical assignment agreement range	63.6%-98.9%
Mean agreement: C3	66.2%
Mean agreement: C1	91.7%
Mean agreement: C2	87.9%
Mean agreement: C5	94.2%
Mean agreement: C4	86.1%

The lower C3 agreement reflects overlap with C1. The clinical distinction was evaluated using baseline burden and response magnitude rather than assumed solely from cluster labels.

Table S9. Omnibus differences among five phenotypes.

Variable	Kruskal-Wallis H	p
Age	26.14	2.96e-05
Baseline BMI	52.67	9.97e-11
KOA duration	20.94	0.000325
Baseline VAS	67.19	8.87e-14
Baseline WOMAC	62.97	6.9e-13

Variable	Kruskal-Wallis H	p
Baseline CRP	52.40	1.14e-10
Baseline ESR	42.55	1.28e-08
Baseline HbA1c	29.85	5.26e-06
Achieved dose	78.03	4.55e-16
Weight loss	71.16	1.29e-14
VAS improvement	69.23	3.3e-14
WOMAC improvement	76.78	8.37e-16
CRP reduction	57.55	9.48e-12
ESR reduction	58.72	5.38e-12
HbA1c reduction	45.09	3.81e-09

Table S10. Focused comparison of C3 and C1.

Characteristic	C3	C1	Mann-Whitney p
Achieved dose, mg	2.40	2.40	Not applicable (identical)
Weight loss, %	13.83	13.62	0.435
Baseline VAS	7.61	7.00	<0.001
Baseline WOMAC	68.26	63.13	<0.001
Baseline CRP, mg/L	7.45	6.54	0.005
Baseline ESR, mm/h	29.00	24.74	<0.001
VAS improvement	3.43	2.91	<0.001
WOMAC improvement	29.48	27.04	<0.001
CRP reduction, mg/L	3.55	3.00	<0.001
ESR reduction, mm/h	9.78	7.65	<0.001
HbA1c reduction	0.67	0.56	0.006

C3 and C1 were retained separately because their exposure and weight loss were similar, while baseline inflammatory/symptomatic burden and multidomain response differed. Their boundary remains less stable than those of the other phenotypes.

Table S11. Within-phenotype correlations between percentage weight loss and selected improvements.

Phenotype	Outcome	Pearson r	p
C3	VAS	0.22	0.306
C3	WOMAC	0.22	0.304
C3	CRP	0.37	0.084
C3	ESR	0.15	0.493
C3	HbA1c	-0.12	0.573
C1	VAS	0.17	0.426
C1	WOMAC	0.01	0.950
C1	CRP	0.45	0.032
C1	ESR	-0.08	0.717
C1	HbA1c	0.13	0.556
C2	VAS	0.35	0.184
C2	WOMAC	0.67	0.004
C2	CRP	-0.63	0.010
C2	ESR	-0.38	0.141
C2	HbA1c	-0.15	0.570
C5	VAS	0.30	0.248
C5	WOMAC	0.30	0.237

Phenotype	Outcome	Pearson r	p
C5	CRP	-0.01	0.963
C5	ESR	-0.05	0.841
C5	HbA1c	0.07	0.786
C4	VAS	0.57	0.110
C4	WOMAC	0.63	0.068
C4	CRP	-0.27	0.481
C4	ESR	0.13	0.739
C4	HbA1c	0.76	0.018

These correlations are descriptive. Small cluster sizes and restricted within-cluster ranges make them unstable and unsuitable for confirmatory inference. The absence of correlation in C5 is hypothesis-generating, not proof of independence.

S6. Safety Laboratory Detail

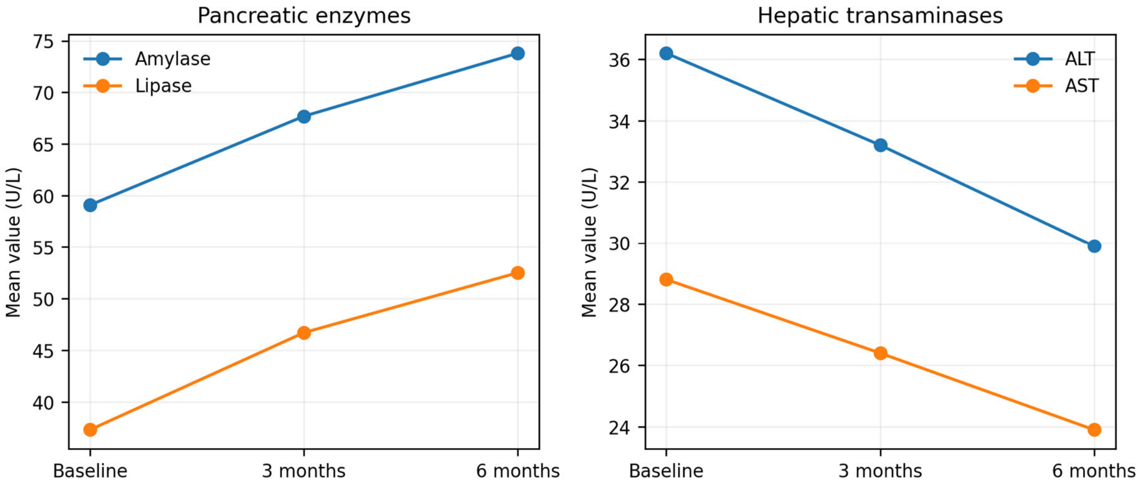

Figure S4. Descriptive pancreatic enzyme and hepatic transaminase trajectories among participants with available measurements.

Table S12. Interpretation of safety trajectories.

Domain	Observed pattern	Interpretation
Pancreatic enzymes	Modest mean increases in amylase and lipase	No clinically suspected pancreatitis was documented; laboratory increases alone are not diagnostic.
Hepatic transaminases	Mean ALT and AST decreased	Compatible with improved metabolic status but not attributable causally.
Clinical events	One abdominal pain report; no pancreatic discontinuation	Reassuring for this cohort, but uncommon-event risk cannot be estimated precisely.
